# A comparison of antenatally and intraoperatively diagnosed cases of placenta accreta spectrum

**DOI:** 10.4274/jtgga.galenos.2019.2019.0063

**Published:** 2020-06-08

**Authors:** Rahila Imtiaz, Zubaida Masood, Samia Husain, Sonia Husain, Rubina Izhar, Saba Hussain

**Affiliations:** 1 Department of Gynaecology and Obstetrics, Karachi Medical and Dental College, Karachi, Pakistan

**Keywords:** Placenta accreta spectrum, antenatal diagnosis, fetomaternal outcomes

## Abstract

**Objective:**

To assess the effect of antenatal diagnosis of placenta accreta spectrum (PAS) on fetomaternal outcomes.

**Material and Methods:**

This was a retrospective cohort study conducted from January 2017 to December 2018. Women with PAS diagnosed antenatally were designated as group A and those where diagnosis was suspected during operation and confirmed on histopathology (PAS diagnosed perioperatively) were designated as group B. Outcome in terms of uterine conservation, maternal death, admission of mother to intensive care unit (ICU), perinatal death and neonatal ICU (NICU) admission were recorded.

**Results:**

During the study, PAS was confirmed in 96 cases which were included. Out of these, 34 (35.4%) cases were included in group A while 62 (64.6%) were diagnosed intraoperatively (group B). The median number of units of blood transfused was lower in group A compared to group B (4 vs 6, p<0.001). The uterus was conserved more often in group A compared with group B (67.6% vs 43.5%, p=0.024) while admission to ICU occurred significantly more often in group B (26.5% vs 59.7%, p=0.002). Maternal death (p=0.038) and perinatal death (p=0.008) were also significantly higher in group B. More neonates delivered to mothers in group B were admitted to NICU (85.7% vs 24%, p=0.033). Survival analysis showed a statistically significant increase in uterine conservation rate in group A compared with group B (log rank, p=0.04).

**Conclusion:**

PAS diagnosed antenatally has better fetomaternal outcome than intraoperative detection of PAS. Diagnosing PAS antenatally is therefore crucial to improve management and achieve a better outcome.

## Introduction

Placenta accreta spectrum (PAS) is a well-known entity that has become far more common than previously reported (1). This is partly due to the rising cesarean section rates in the region. However, the effect of sophisticated techniques for diagnosing this condition is also noteworthy.

PAS is associated with significant maternal morbidity and mortality. The condition, when diagnosed antenatally, allows mobilization of suitable clinical resources and helps to reduce poor outcomes (2). It was previously believed that the final diagnosis could only be confirmed retrospectively by histological examination of the specimen. This position is now in doubt and studies have reported that novel techniques such as power Doppler and magnetic resonance imaging have up to 100% sensitivity in diagnosing PAS cases (3).

An urgent problem arises when PAS cases are diagnosed intrapartum and the expertise, though available at tertiary centers, cannot be mobilized rapidly enough (4). Diagnosis of PAS can be dependent on assessment of risk factors but this is not always sufficient so that sometimes cases are missed, especially in facilities with high patient workloads. When an undiagnosed PAS is encountered morbidity has been reported to increase (5).

Despite antenatal diagnosis of PAS being associated with decreased morbidity, the evidence remains sparse, as PAS is not frequently encountered worldwide. Most evidence has been gathered in regions where antenatal care is optimal and cesarean sections rates are not very high (6,7). This report originates from a region where antenatal care is suboptimal and cesarean section rates are at an all-time high which results in cases of PAS being seen more frequently. This aim of this study was to assess the effect of antenatal diagnosis of PAS on morbidity seen in such cases.

## Material and Methods

This was a retrospective cohort study, conducted from 1^st^ January 2017 to 31^st^ December 2018. Consent for the use of hospital records was obtained from the department head. All labor room and obstetrics theatre records were analyzed to calculate the delivery rate at the hospital. The incidence of accreta was then calculated for the facility. All women who were diagnosed with PAS on histopathology report were included in the analysis. Women whose histopathology was not sent and PAS was not confirmed were excluded. All cases were scrutinized for diagnosis; women who were diagnosed before they underwent anesthesia for cesarean delivery were included as antenatally diagnosed PAS (group A). Cases where PAS was suspected during operation and confirmed on histopathology were included as PAS diagnosed peroperatively (group B).

For cases diagnosed antenatally, the surgery was performed by a senior obstetrician and a consultant anesthetist while a consultant pediatrician was present. Four units of blood were arranged and continuous communication was maintained with the onsite blood bank. The patient’s hemoglobin was regularly assessed antenatally and hemoglobin of above 11 g/dL was maintained. All patients received antenatal steroids to promote lung maturation in the fetus after 26 weeks. For this purpose, injection betamethasone (Betnesol) was used at a dose of 12 mgs. Each patient was given two doses, intramuscularly, 24 hours apart. The incision was made avoiding the placental location, which was assessed preoperatively by an ultrasound assessment. The baby was delivered by going around the placenta and was immediately handed over to the consultant pediatrician for ongoing neonatal care.

A proforma was used to collect data that included: Age of woman, her parity, duration of surgery in minutes and number of units of blood transfused. The primary outcome measure was uterine conservation. Secondary outcome measures included maternal death, admission of mother to intensive care unit (ICU), perinatal death or neonatal ICU admission. Duration of surgery in cases where the uterus was conserved was also assessed.

Data was coded and confidentiality was ensured. The hospital head gave permission to the investigators for reporting the study. In lieu of formal ethical approval, the principles of the Declaration of Helsinki were followed.

### Statistical analysis

All data were analyzed using SPSS, version 15 (IBM Inc., Chicago, IL, USA). Shapiro Wilk’s test was used to assess the normality of data. Women’s age, parity, duration of surgery and number of units transfused were not normally distributed and were presented as median and range. Mann-Whitney U test was used to compare non-parametric data sets. Frequencies and percentages were calculated for qualitative variables including uterine conservation, diagnosis antepartum/intrapartum, ICU admission and neonatal death. Chi-square test and Fisher’s exact test were used to compare these variables at p<0.05 level of significance.

Time to uterine conservation was analyzed for both groups using the Kaplan-Meier survival plot and curves were compared by means of Mantel Haenszel log rank test. A significance level of 5% was chosen.

## Results

During the study period, 8979 deliveries took place at the facility. PAS was confirmed in 96 of those women, giving an incidence of 1.06% (1 in 100). Of these, 34 (35.4%) cases were antenatally diagnosed (group A), while 62 (64.6%) were diagnosed intraoperatively (group B).

The median (range) age of the study population, time of surgery and number of units of blood transfused was 28 (21-35) years, 55 minutes (50-140) minutes and 6 (2-12) units, respectively. Only 34 (35.4%) cases were diagnosed antepartum. When risk factors were assessed, the median (range) number of previous section in the study population was 1 (0-4) and only 16 (16.7%) had a history of dilation and curettage while 40 (41.7%) gave history of bleeding. Uterus was conserved in 50 (52.1%) of all women. ICU admission was required for 46 (47.9%) of women and 23 (23.59%) women died. Regarding perinatal outcomes, perinatal mortality rate was 44.8%. Of these, 31 (72.09%) were fresh stillbirths and 12 (27.91%) were neonatal deaths. Only 18 (33.96%) neonates had Apgar score below 7 after 5 minutes and ICU admission was required for 30 (56.6%) of the neonates. Table 1 summarizes the characteristics of the whole study population.

When stratified according to groups, there was no significant difference between the groups with regards to age (p=0.865), parity (p=0.289) and duration of surgery (p=0.588). There was no difference between the groups in terms of risk factors including the median number of cesarean sections (p=0.304), history of bleeding (p=0.703) and history of prior dilatation and curretage (p=0.427). However, antenatally diagnosed PAS required a lower median number of units of blood transfused compared to those with intrapartum diagnosed PAS (4 vs 6, p<0.001).

Uterus was conserved in 23 (67.6%) women in group A and 27 (43.5%) women in group B (p=0.024). Nine (26.5%) women required admission to ICU in group A which was significantly fewer (p=0.002) than the 37 (59.7%) of women from group B who required ICU care. Maternal death (p=0.038) and perinatal death (p=0.008) were also significantly more frequent in cases diagnosed perioperatively (p=0.008). More neonates delivered to women in group B (85.7% vs 24%, p=0.033) were admitted to ICU for cases diagnosed intraoperatively (see Table 2).

**Figure 1:**
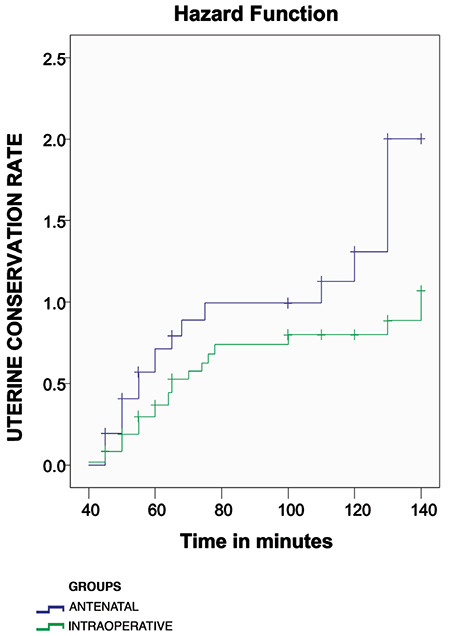
Kaplan meier survival curve of women whose uteri were conserved (log rank test, p=0.04)

Survival analysis showed a statistically significant difference in the duration of surgery with antenatal diagnosis between the two groups (log rank, p=0.04; see Figure 1).

## Discussion

### Main findings

The present study shows that feto-maternal outcomes are better in cases of PAS where diagnosis is made antenatally.

Women need fewer units of blood when diagnosed antenatally and are more likely to retain their uterus. Duration of surgery is also shorter in antenatally diagnosed cases of PAS and there is a significantly lower likelihood of admission to ICU following surgery while perinatal death is also less likely.

### Study Limitations

As placenta accreta is seen frequently due to higher cesarean rates, the strength of this study is its sample size. Another strength of the study is the clear clinical definition of PAS as all cases that were included were histologically confirmed.

The biggest limitation of the study was the retrospective design. As cases were assessed retrospectively, patient reported outcomes could not be assessed.

### Interpretation

Rates of placenta accreta have increased markedly in recent times. Placenta accreta was previously only seen occasionally. In developed countries the rates of PAS are lower than those reported from developing countries. The estimated incidence from the UK was 1.7 per 10000 maternities (8) while that from Australia was 44.2/100000 women giving birth (9). The prevalence varied from 0.01% to 1.1% according to a large database review (10). These rates are alarming as proportion of cesarean sections of all deliveries is also increasing and this could potentially mean more cases would be encountered in the near future. The rate in our study was 0.010% which is very high in comparison to the data previously reported. Another reason for this rate could be the fact that the facility is a tertiary care center and deals with all high-risk cases that are referred from all parts of the city.

Increased maternal age, previous cesarean section and increased parity are among the commonly recognized risk factors for PAS (11). The median age of the study population was 28 years and they had a median parity of 2 which shows that the women were below the advanced maternal age range and might have not completed their families. The median maternal age reported for placenta accreta was approximately 34 years and the median parity was 2.5 (12). This suggests that placenta accreta may soon affect younger women, as PAS in the most commonly reported indication of peripartum hysterectomy.

Antenatal diagnosis of PAS can substantially reduce morbidity. Only 35.4% of our series were diagnosed antepartum. This is lower than the 57% of cases diagnosed antenatally in a study reported from Australia (9). In that study, 36% of the cases were diagnosed solely on ultrasound. Our hospital records showed that all cases of PAS that were antenatally diagnosed, were suspected on grey scale ultrasound and confirmed on color Doppler. 3-D power Doppler has been shown to have the best prediction of antenatal PAS (13). Since 3-D power Doppler was not available at our center, our cases were diagnosed by color Doppler.

Availability of senior colleagues or experts has been shown to improve outcome in difficult and complex cases (14). Multidisciplinary team involvement and specialized centers for managing PAS have been reporting better outcomes. However, these resources can only be mobilized in antenatally diagnosed patients. In cases where accreta is diagnosed on the operating table, such expertise is difficult to arrange at very short notice (15). Our study highlights the importance of preparedness for such emergencies as antenatally diagnosed cases had significantly better outcomes.

The duration of surgery is an important morbidity marker. Women with prolonged surgeries are more likely to require ICU transfer, have a longer duration of stay in hospital, and suffer wound infections and other post-operative complications (16). Duration of surgery was not significantly different between the groups in our study but women who had PAS diagnosed antenatally had a lower transfused blood volume requirement. In addition, in cases where the uterus was conserved, time to uterine conservation was also less. This finding may be explained by the fact that these cases were optimized antenatally, hemoglobin was kept in check and the operation was performed by more experienced surgeons under more favorable conditions. The planning and anticipation of complications in cases of PAS is associated with improved outcomes overall (17).

Fetomaternal outcomes were also influenced by the timing of diagnosis in the study. Only 26.5% of group A compared with 59.7% of group B were admitted to ICU which was a significant difference (p=0.002). Perinatal death was also significantly higher in cases diagnosed intrapartum (p=0.008). These findings are in agreement with the studies reported previously (18,19).

Our study shows the beneficial effect of diagnosing PAS antenatally. This diagnosis becomes even more important in regions where antenatal care remains suboptimal because these women have far worse fetomaternal outcomes than women who receive better care. The importance of a scan to localize the placenta antenatally in all women and confirm invasion, especially in cases with a previous scar, cannot be emphasized enough.

Simply diagnosing PAS is not sufficient, in our opinion. We believe there is a need for dedicated centers for PAS. Women in our study, despite delivering at a tertiary care center, did not achieve as good an outcome as reported by studies from the developed world.

If PAS is diagnosed antenatally, women are counseled and optimized prior to delivery and delivery subsequently takes place in a dedicated center with sufficient expertise, much better outcomes can be expected. Development of dedicated PAS centers in developing countries can be expected to make a significant difference in reducing morbidity and both maternal and fetal mortality. After establishment of such centers, a larger study with prospective design would be capable of assessing the outcomes with better and more prevalent antenatal diagnosis.

## Conclusion

PAS diagnosed antenatally has better fetomaternal outcomes than intraoperative detection of PAS. Our results show that diagnosing PAS antenatally will significantly improve management and result in better outcome. We propose adoption of antenatal localization of placenta in all cases, especially where risk factors for PAS are present.

## Figures and Tables

**Table 1:**
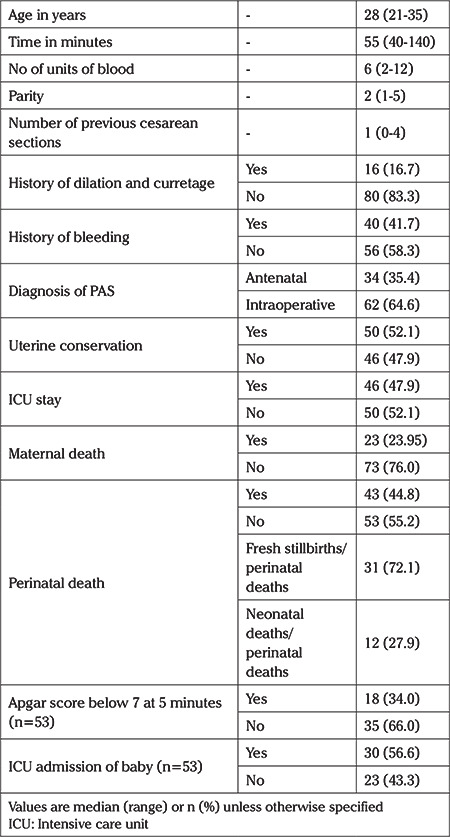
Demographic and clinical characteristics of the whole study population

**Table 2:**
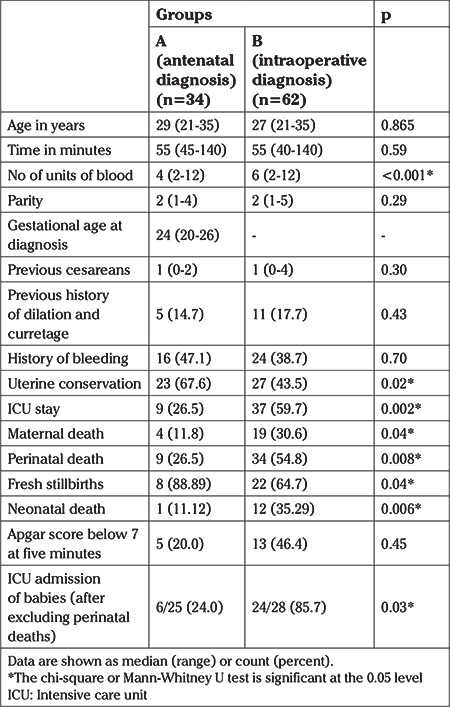
Demographic and clinical characteristics by group
